# Lipidomic Analysis of Endocannabinoid Signaling: Targeted Metabolite Identification and Quantification

**DOI:** 10.1155/2016/2426398

**Published:** 2015-12-29

**Authors:** Jantana Keereetaweep, Kent D. Chapman

**Affiliations:** ^1^Department of Biological Sciences, Center for Plant Lipid Research, University of North Texas, Denton, TX 76203, USA; ^2^Brookhaven National Laboratory, 50 Bell Avenue, Building 463, P.O. Box 5000, Upton, NY 11973-5000, USA

## Abstract

The endocannabinoids *N*-arachidonoylethanolamide (or anandamide, AEA) and 2-arachidonoylglycerol (2-AG) belong to the larger groups of *N*-acylethanolamines (NAEs) and monoacylglycerol (MAG) lipid classes, respectively. They are biologically active lipid molecules that activate G-protein-coupled cannabinoid receptors found in various organisms. After AEA and 2-AG were discovered in the 1990s, they have been extensively documented to have a broad range of physiological functions. Along with AEA, several NAEs, for example, *N*-palmitoylethanolamine (PEA), *N*-stearoylethanolamine (SEA), and *N*-oleoylethanolamine (OEA) are also present in tissues, usually at much larger concentrations than AEA. Any perturbation that involves the endocannabinoid pathway may subsequently alter basal level or metabolism of these lipid mediators. Further, the altered levels of these molecules often reflect pathological conditions associated with tissue damage. Robust and sensitive methodologies to analyze these lipid mediators are essential to understanding how they act as endocannabinoids. The recent advances in mass spectrometry allow researchers to develop lipidomics approaches and several methodologies have been proposed to quantify endocannabinoids in various biological systems.

## 1. Introduction

Since the discovery of the cannabinoid receptors [1] and their endogenous ligands [2, 3], considerable progress has been made in the understanding of the endocannabinoid system and its role as a lipid signaling pathway which modulates several physiological processes and pathological conditions. Several studies have shown that levels of some major endocannabinoids are altered in various tissue systems [4–7]. The development of sensitive and accurate methodologies to analyze endocannabinoid levels will lead to a more detailed understanding of the role of these lipid metabolites in regulating mammalian physiology.

**Figure 1:**
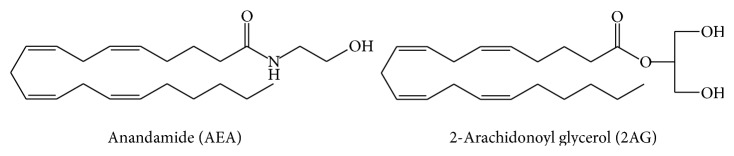
Structures of AEA and 2-AG glycerol, the two best known endocannabinoids.

The two most documented endocannabinoids, AEA and 2-AG, are derivatives of arachidonic acid (Figure 1) which were isolated originally from porcine brain [2] and canine intestine [3] in the early 1990s. They belong to larger classes of lipids which are* N*-acylethanolamines (NAEs) and monoacylglycerols (MAGs), respectively, most of which do not bind with high affinity to cannabinoid receptors. AEA has been reported to elicit similar biological effects to tetrahydrocannabinol (THC), a potent bioactive secondary metabolite in marijuana and in rodents [8] as well as in humans [9]. 2-AG has also been reported to have wide range of neurological functions (e.g., synaptic plasticity and neuroprotection, as relevant to this special issue) [10]. AEA is released from* N*-arachidonyl phosphatidylethanolamine on demand via the hydrolytic activity of* N*-acylphosphatidylethanolamine (NAPE) phospholipase D (NAPE-PLD) [11], although other routes of synthesis have been described [12–14]. The presence of a two-step pathway also was reported, which involves the hydrolysis of one* O*-acyl moiety of NAPE to form N-acyl-lyso PE by phospholipase A (PLA1 or PLA2), and the subsequent release of AEA from N-acyl lysoPE by PLD [15]. Another alternative pathway of AEA formation was also reported in mouse brain and RAW264.7 macrophages which comprises the hydrolysis of NAPE by phospholipase C (PLC) to form phosphoanandamide (pAEA), followed by dephosphorylation by phosphatases including previously described tyrosine phosphatase PTPN22 [13]. On the other hand, 2-AG is synthesized by PLC and diacylglycerol-lipases (DAGL) [16]. Both AEA and 2-AG are inactivated by hydrolysis of the amide and ester bonds, respectively. Fatty acid amide hydrolase (FAAH) catalyzes the hydrolysis of AEA [17] while monoacylglycerol lipase (MAGL) is responsible for hydrolysis of 2-AG [18].

Schmid et al. reported that the relative abundance of different NAE species in animal cells generally reflects the acyl groups in the* N*-acylphosphatidylethanolamine (NAPE) precursor which is a minor membrane lipid component [20]. In fact, the first NAEs discovered in mammalian tissues were* N*-palmitoylethanolamine (PEA, NAE 16 : 0) and* N*-stearoylethanolamine (SEA, NAE 18 : 0) which were reported to be endogenous components in brains and peripheral tissues of rats and guinea pigs [21, 22] and now have been shown to be widespread in occurrence. However, PEA and SEA are mostly considered to be CB receptor-inactive because of their limited binding to CB receptors. AEA is the most studied of the NAEs due to its ability to bind to and activate endocannabinoid CB_1_ and CB_2_ receptors, and in the endocannabinoid signaling system, AEA activity has been proposed to be influenced by the presence of other receptor-inactive NAEs as a sort of “entourage effect,” since all NAEs compete for the same turnover machinery [22]. Other studies showed the accumulation of receptor-active and receptor-inactive NAEs (mainly* N*-palmitoylethanolamine,* N*-stearoylethanolamine,* N*-oleoylethanolamine (OEA, NAE 18 : 1), and* N*-linoleoylethanolamine (NAE 18 : 2) [23]) in infracted dog heart muscle. Accumulation of NAEs has been associated with stress and tissue damage in other systems, such as in the circulation of human during acute stress, damaged human epidermal cells, and focal cerebral ischemia in mice [24–26], just to name a few.

The oxidative products of polyunsaturated NAEs, including eicosanoid ethanolamides, prostaglandins, and leukotrienes, are believed to be important signaling compounds that participate in diverse physiological processes [27, 28]. Recent studies showed that AEA can be oxidized to prostaglandin ethanolamides (prostamide) by cyclooxygenase-2 (COX-2) [29–31]. The first prostamide discovered was PGE_2_-ethanolamide [32]. The pharmacological effects of the prostamide F_2*α*_ and its analogue bimatoprost have been studied extensively, especially with respect to the antiglaucoma properties of bimatoprost [33–35]. AEA has also been reported to serve as a substrate for 12-LOX and 15-LOX in human polymorphonuclear leukocytes and human platelets, generating 12- and 15-hydroperoxyeicosatetraenoylethanolamide (12-HETE-EA and 15-HETE-EA), respectively [36]. Oxidative metabolites of AEA generated by 12-LOX were proposed to play roles in pain modulation by acting as a vanilloid receptor agonist [37], while 15-LOX product showed inhibition of electrically evoked contraction of mouse vas deferens by acting as a cannabinoid receptor agonist [38]. In addition to metabolism by COX-2 and LOXs, AEA also undergoes oxidation by several of human cytochrome P450 enzymes such as CYP3A4, CYP4F2, and CYP4X1, resulting in various oxidized lipid species, some of which may have biological relevance [39]. For example, there is evidence that a cytochrome P450-derived epoxide of AEA can act as a potent agonist of CB_2_ receptors.

**Figure 2:**
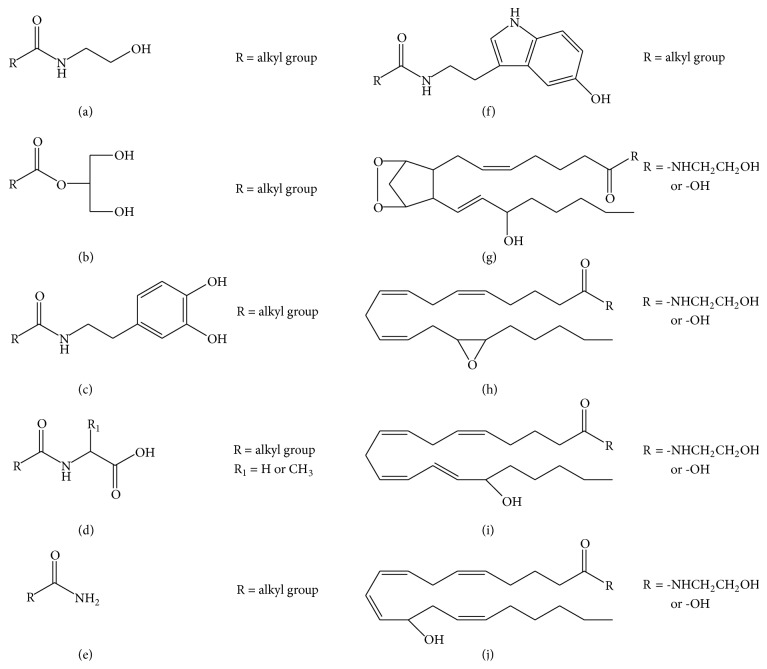
Chemical structures of different classes of endocannabinoids and endocannabinoid-like molecules reported. (a)* N*-Acylethanolamine, (b) monoacylglycerol, (c)* N*-acyldopamine, (d)* N*-acyl amino acid, (e)* N*-acylamine, (f)* N*-acylserotonin, (g) COX-2 derivatives, (h) CYP450 derivatives, (i) 15-LOX derivatives, (j) and 12-LOX derivatives.

Other than oxidative derivatives, unsaturated fatty acid conjugates with various amines/neurotransmitters have been reported, some with cannabinoid-like or vanilloid-like activity [40]. For example,* N*-arachidonoyl dopamine (NADA), the first member of the* N*-acyldopamine family, was reported to have capsaicin-like activity and to activate the transient receptor potential vanilloid-1 (TRPV1) receptor with similar potency and efficacy to capsaicin [41].* N*-Arachidonylglycine was found in bovine and rat brain and was shown to suppress tonic inflammatory pain [42]. In addition, a member of* N*-acylserotonin family of conjugates,* N*-arachidonoyl serotonin (AA-5-HT), was shown to be highly effective against both acute and chronic peripheral pains and exhibited FAAH inhibition and TRPV1 antagonist activities [43]. Although not an arachidonic acid conjugate, a structurally related fatty acid amide, oleamide, a primary amide of oleic acid, was reported by Cravatt et al. to be a potent, endogenous sleep inducing lipid in mammals [44]. Several groups of endocannabinoids and endocannabinoid-like molecules are illustrated in Figure 2.

Due to increasing interest in the biological significance of endocannabinoids and endocannabinoid metabolites, several lipidomics profiling approaches have been developed to identify and quantify endocannabinoids in various biological tissues. Lipidomics techniques discussed here include the separation and quantification of endocannabinoid-like and endocannabinoid-like compounds by gas chromatography-mass spectrometry (GC-MS) and liquid chromatography-mass spectrometry (LC-MS), as well as methods for derivatization and detection of these metabolites for identification, especially focused on quantitative methodologies for AEA, 2-AG, and other NAE metabolites.

## 2. Extraction of Endocannabinoids from Biological Samples

### 2.1. Tissue Homogenization

The ultimate success in quantification of trace endocannabinoid metabolites is dependent in a large part on processes for handling of tissues and extraction of metabolites [45]. Extraction of endocannabinoid from biological samples typically includes tissue homogenization in organic solvent followed by protein precipitation and lipid extraction. Tissues to be analyzed are snap-frozen in liquid nitrogen or cold 2-methylbutane and kept in −80°C before analysis. Prior to homogenization, frozen tissues should be quickly weighed without thawing. Alternatively, to limit any chance of postmortem changes in metabolite amounts that may be generated during thawing, lipids can be extracted before thawing and dry weights of tissue residues following lipid extraction can be used for normalization, since most tissues contain less than a few percent lipid of the total tissue dry weight. The homogenization of tissue can be achieved by using different methods, including, but not limited to, silanized glass homogenizer [46], electric homogenizer [47–49], or bead beater with glass beads [50]. The homogenization step is usually performed rapidly and on ice to prevent degradation of analytes. A known amount of an internal standard is also added in the beginning of tissue homogenization for quantification purposes.

### 2.2. Internal Standards (IS) for Quantification

**Figure 3:**
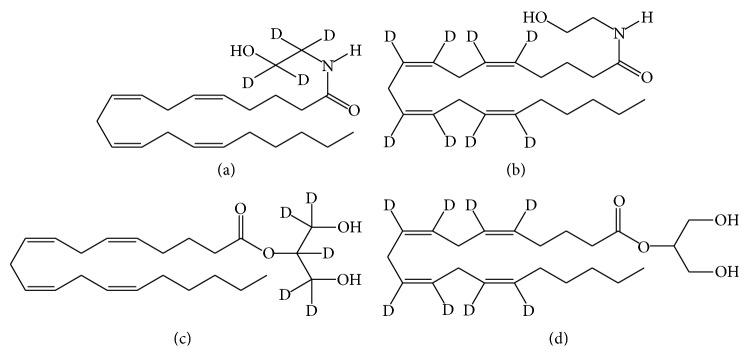
Commercially available internal standards for analysis by mass spectrometry. (a) Arachidonoyl ethanolamide-d_4_ (AEA-d_4_). (b) Arachidonoyl ethanolamide-d_8_ (AEA-d_8_). (c) 2-Arachidonoyl glycerol-d_5_ (2-AG-d_5_). (d) 2-Arachidonoyl glycerol-d_8_ (2-AG-d_8_).

Standard curve (calibration curve) construction is a critical step to ensure accuracy, reproducibility, and reliability when establishing analytical methods for quantifying metabolites in extracts from biological samples. An internal standard approach requires that a constant amount of nonendogenous or nonanalyte molecules is added to each sample prior to homogenization. Internal standards must behave similarly to analytes throughout the extraction procedures, and recovery should be on the order of 90% or better. In MS-based analysis, stable-isotope labeled internal standards are ideal due to the difference in atomic mass units (amu) between internal standards and analytes (Figure 3). For GC, UV, or fluorescence-based analysis, internal standards with different carbon chain length, for example, heptadecanoyl ethanolamide [51] can be utilized. Two stable-isotope labeled AEA analogs, AEA-d4 and AEA-d8, are commercially available. In AEA-d4, the four deuterium atoms are located on the ethanolamine moiety while eight deuterium atoms in AEA-d8 are located on the carbon atoms that form the double bonds of arachidonic acid. For 2-AG, 2-AG-d8 and 2-AG-d5 are commercially available. In 2-AG-d5, the five deuterium atoms are located on the glycerol moiety while eight deuterium atoms in 2-AG-d8 are located on the carbon atoms that form the double bonds of arachidonic acid (Figure 3) [19, 52].

### 2.3. Protein Precipitation

Protein precipitation step is commonly performed prior to lipid extraction, especially in LC-based analysis of endocannabinoids. Acetone is frequently used in this step [53–55]. Other water-miscible solvents such as acetonitrile [56, 57] or methanol [58] also efficiently precipitate the proteins. Supernatant is subsequently collected and subjected to further lipid extraction.

### 2.4. Lipid Extraction

The most widely used methods for endocannabinoid extraction from various tissues are based on the classic total lipid extraction described by Folch et al. [59] or Bligh and Dyer [60]. Due to the lipophilic nature of endocannabinoids, they are usually extracted from biological samples using combinations of water-immiscible solvent. The mixture of chloroform/methanol (or 2-propanol) of different ratios is frequently used [47, 53, 61–63]. Small amounts of water can also be added to create phase separation. Instead of chloroform/methanol, some groups also used chloroform alone, ethyl acetate/hexane mixture [46], or methanol [64]. Repeated extraction usually significantly increases lipid yield. One of the simplest methods developed by the Hillard group is to sonicate tissue in acetonitrile prior to protein precipitation at −10°C. This method was shown to result in relatively clean extract suitable for LC-based analysis without any further analyte enrichment steps [65, 66].

Still, the relative abundance of endocannabinoids is low compared with that of structural membrane lipids or storage lipids in biological samples. High levels of matrices can compromise the analysis by causing ion suppression or interfering with signals in the MS or analyte recovery. Thus, the optimization of endocannabinoid analysis often requires further purification of the extracts. This can be achieved by an endocannabinoid enrichment step using thin layer chromatography (TLC) or solid phase extraction (SPE).

### 2.5. Endocannabinoid Enrichment from Crude Extracts

#### 2.5.1. Thin Layer Chromatography (TLC)

Concentrated lipid extracts in chloroform/methanol are spotted onto TLC plate. The plate is subsequently developed in various solvent combinations, for example, chloroform/methanol [67], chloroform/methanol/ammonia [67], chloroform/hexane/methanol [51], ethyl acetate/water/2,2,4-trimethylpentane/acetic acid [68], or petroleum ether/diethyl ether/acetic acid [69]. After separation, the spot (or band) containing endocannabinoids scraped off and extracted with organic solvent such as chloroform/methanol for subsequent analysis.

#### 2.5.2. Solid Phase Extraction (SPE)

The SPE approach is more frequently used than TLC. Unlike TLC, SPE can be fully automated which is more suitable for high-throughput analysis. SPE is convenient and can be applied for isolation of analytes from a variety of matrices. SPE used for lipid metabolite enrichment can be divided into two categories, reverse phase and normal phase.

*(1) Reverse Phase SPE*. Reverse phase SPE columns contain matrices that are comparatively more lipophilic, for example, C8 or C18. The columns are usually activated with methanol and conditioned with water prior to loading of lipid extracts suspended in polar solvent (e.g., methanol/water). After sample loading, endocannabinoids are eluted from the column with methanol [47, 70, 71] or 1 : 1 v/v cyclohexane/ethyl acetate [70]. Columns also can be washed with 20% v/v acetonitrile in water with endocannabinoid metabolites eluted in 80% v/v acetonitrile in water containing 0.1% TFA [57]. Multiple washes of the column with solvent may be required to remove contaminants from the chromatography matrices. The eluates with analytes from SPE may be dried under a nitrogen gas stream prior to derivatization.

*(2) Normal Phase SPE*. Normal phase SPE columns contain a more hydrophilic matrix, for example, silica gel. Silica-gel SPE columns can be made in the laboratory by using Pasteur pipettes plugged with a small piece of glass wool and filled with slurry of silica and chloroform [47, 63]. There are also several brands and sizes commercially available. The columns are normally washed with chloroform prior to loading of lipid extracts also in chloroform. The column is subsequently washed again with chloroform. The ethanolamine moiety of NAEs and glycerol moiety of MAGs interact with silanol groups in the column which results in retention of endocannabinoid metabolites in the column during this step. The endocannabinoid metabolites are eluted from normal phase silica-gel SPE columns with 1 : 1 v/v of ethyl acetate/acetone [47] or 2% v/v methanol in chloroform [72]. A small amount of triethylamine (TEA) and trifluoroacetic acid (TFA) in SPE solvent system was found to improve recovery of analytes [46]. According to Hardison et al., silica-gel SPE can lead to poor recovery and loss of deuterium atoms from internal standards while C18 SPE offered higher recovery and more reproducible MS-based quantification [47].

## 3. Separation and Quantification of Endocannabinoid Metabolites

### 3.1. Gas Chromatography-Mass Spectrometry (GC-MS)

Gas chromatography (GC) has been widely used to separate endocannabinoid metabolites in lipid extracts from biological samples. GC is most often coupled with MS for sensitive detection and quantification of the analytes. Columns normally equipped on CG are nonpolar stationary phase capillary columns, for example, Rtx-5MS (cross-linked diphenyl dimethyl polysiloxane) [47], CP-Sil 8 CB (5% phenyl groups in the dimethylpolysiloxane polymer) [63], DB-1 dimethylpolysiloxane [73], and HP-5/DB-5 (5% phenyl-methylpolysiloxane) [74, 75]. Helium is commonly used as a carrier gas, although hydrogen or nitrogen can be acceptable. For identification, the full mass spectrum of the analyte can be matched to the mass spectrum of the corresponding standard. For improved quantitative sensitivity, diagnostic fragment ions of the analytes can be detected via selected ion monitoring (SIM). Modes of ionization include electron impact (EI) ionization [63, 76], positive chemical ionization (PICI) [47, 76, 77], and negative chemical ionization (NICI) [52, 78, 79].

#### 3.1.1. Nonderivatized Metabolites

When AEA was first reported as a potential ligand for the cannabinoid receptor in porcine brain, Devane et al. established the structure of AEA by using various techniques in MS including direct-exposure chemical ionization (iso-butane-DCI) and electron impact ionization (El). They also showed that both nonderivatized and trimethylsilyl- (TMS-) derivatized AEA showed similar, predictable fragmentation patterns [2]. Two years later, Mechoulam et al. reported the isolation of a second type of cannabinoid receptor ligand, 2-AG, in canine intestine. Here, results with both CI-MS and EI-MS demonstrated expected fragmentation patterns for both nonderivatized and TMS-derivatized 2-AG [3]. Other groups also analyzed AEA and 2-AG in their native forms. Maccarrone et al. [80] analyzed AEA together with endocannabinoid-like NAEs including* N*-oleoylethanolamine,* N*-palmitoylethanolamine, and* N*-stearoylethanolamine in extracts from human brain without derivatization. By using GC-EI-MS with SIM mode, the analytes were quantified against deuterated (D4)-AEA. The limit of detection was reported to be 20 ± 10 pmol [80]. GC/CI-MS also was reported to efficiently detect AEA and endocannabinoid-like compounds (*N*-oleoylethanolamine,* N*-palmitoylethanolamine,* N*-stearoylethanolamine,* N*-linoleoyl ethanolamine,* N*-oleoylpropanolamine, and* N*-palmitoylpropanolamine). [M + H]^+^ ions were used to identify these compounds using a polyethylene glycol phased column and fused silica capillary pretubing [81].

#### 3.1.2. Derivatization of Endocannabinoid Metabolites for GC-MS and GC-MS/MS Analysis

In order to gain more sensitivity, analysis of endocannabinoid metabolites in extracts from biological samples usually requires a chemical derivatization step before separation on GC column. Derivatizing agents give analytes thermal stability and volatility which are suitable for GC-based analysis. AEA and 2-AG both have free hydroxyl group(s) where various derivatizing agents can react and subsequently form ester or ether derivatives. In addition to a hydroxyl group, AEA also has an amide group which was reported to be derivatized by certain compounds (see below for pentafluorobenzoyl chloride [78]).

**Figure 4:**
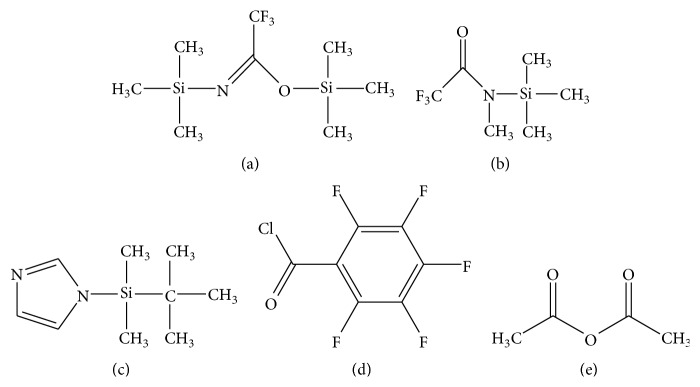
Structures of common derivatizing agents used in GC-based analysis. (a)* N,O*-Bis(trimethylsilyl)trifluoroacetamide (BSTFA). (b)* N*-Methyl-*N*-trimethylsilyl-trifluoroacetamide (MSTFA). (c)* tert*-Butyl dimethylsilyl (*t*BDMS). (d) Pentafluorobenzoyl chloride (PFBzCl). (e) Acetic anhydride.

*(1) Silylation Using N,O-Bis(trimethylsilyl)trifluoroacetamide*. Trimethylsilyl (TMS) derivatives of AEA and 2-AG can be generated by reaction with* N,O*-bis(trimethylsilyl)trifluoroacetamide (BSTFA; Figure 4) which modifies their hydroxyl groups. The reaction is usually carried out at 55°C for 15–50 minutes but can proceed at room temperature as well. Efficiency of silylation is sometimes improved with warming, depending on the target analyte, and so the time and temperature conditions should be empirically determined. The reaction is terminated when BSTFA is completely evaporated under nitrogen stream, although removal of BSTFA is not absolutely required. TMS derivatives typically are dissolved in hexane for analysis by GC-MS. Diagnostic ions of TMS derivatives generated by EI-MS include the [M]^+^ (molecular ion), [*M* − 15]^+^ (loss of one methyl group), and [*M* − 90]^+^ (loss of trimethylsilanol group) [63, 76, 77]. Typical mass spectra generated by chemical ionization MS with positive ion detection (PICI) include TMS-AEA [*M* + H]^+^ (protonated molecule) =* m/z* 420.3, TMS-AEA [*M* + H − 16]^+^ (loss of methane) = 404.3, TMS-AEA [*M* + H − 90]^+^ (loss of trimethylsilanol group) = 330.3, and TMS-2-AG [*M* + H − 18]^+^ (loss of water) = 433.3 [47].

*(2) Silylation Using N-Methyl-N-trimethylsilyl-trifluoroacetamide*. Similarly to silylation with BSTFA, AEA and 2-AG can be converted to their respective TMS derivatives by reacting with* N*-methyl-*N*-trimethylsilyl-trifluoroacetamide (MSTFA; Figure 4) and 1% trimethylchlorosilane (TMCS) at room temperature for 2 h. Detected diagnostic ions generated by EI-MS are as follows: TMS-AEA [M]^+^ =* m/z* 419, TMS-AEA [M − CH_3_]^+^ =* m/z* 404, TMS-TMS-2-AG [M]^+^ =* m/z* 522, and TMS-TMS-2-AG [M − CH_3_]^+^ =* m/z* 507 from [82].

*(3) Silylation Using tert-Butyl Dimethylsilyl (tBDMS) Imidazole*. AEA and 2-AG can be derivatized with* tert*-butyl dimethylsilyl (*t*BDMS) imidazole (Figure 4) at 80°C for 1 h. When* t*BDMS derivatives of these compounds are dissolved in hexane and analyzed by GC-(EI)MS, the characteristic ions* m/z *[M − tert-butyl]^+^ were generated. The ion* m/z* 404 for* t*BDMS-AEA [M − tert-butyl]^+^ [72] and ion* m/z* 549 for* t*BDMS-*t*BDMS-2-AG [M − tert-butyl]^+^ [69] can be monitored.

*(4) Acylation Using Pentafluorobenzoyl Chloride*. AEA possesses a hydroxyl group and an amide group and can be derivatized with pentafluorobenzoyl chloride (PFBzCl; Figure 4) to form* bis-*pentafluorobenzoyl (PFB2) ester derivative. The reaction is carried out at 80°C for 90 minutes using PFBzCl and 4% pyridine in toluene. In NICI, the molecular anion (PFB2-AEA [M]^−^) can be observed at* m/z* 735 [78].

*(5) Acetylation Using Acetic Anhydride*. AEA can be derivatized with acetic anhydride (Figure 4) in pyridine overnight. The reaction occurs at room temperature and is terminated with excess methanol. Full mass spectra of* O*-acetylated-AEA obtained by GC/EI-MS yield the molecular ion (CH_3_CO-AEA [M]^+^), loss of CH_3_CO ([M − 43]^+^), loss of CH_3_COOH ([M − 60]^+^), loss of CH_3_COO(CH_2_)_2_NH ([M − 102]^+^), and loss of CH_3_COO(CH_2_)_2_NHCO ([M − 130]^+^) [67].

### 3.2. Liquid Chromatography

In liquid chromatography- (LC-) based analysis of endocannabinoid metabolites, the stationary phase is usually reverse phase but normal phase LC has also been reported [83]. Various combinations and ratios of mobile phase solvents were used including methanol/water [53, 61, 67, 70, 84], acetonitrile/water [62, 85], water/acetonitrile/isopropanol [71], and methanol/water/acetonitrile [57]. Detectors coupled with LC for detection and/or quantification can be UV, fluorescence, or mass selective detectors.

#### 3.2.1. HPLC with UV and Fluorescence Detection

**Figure 5:**
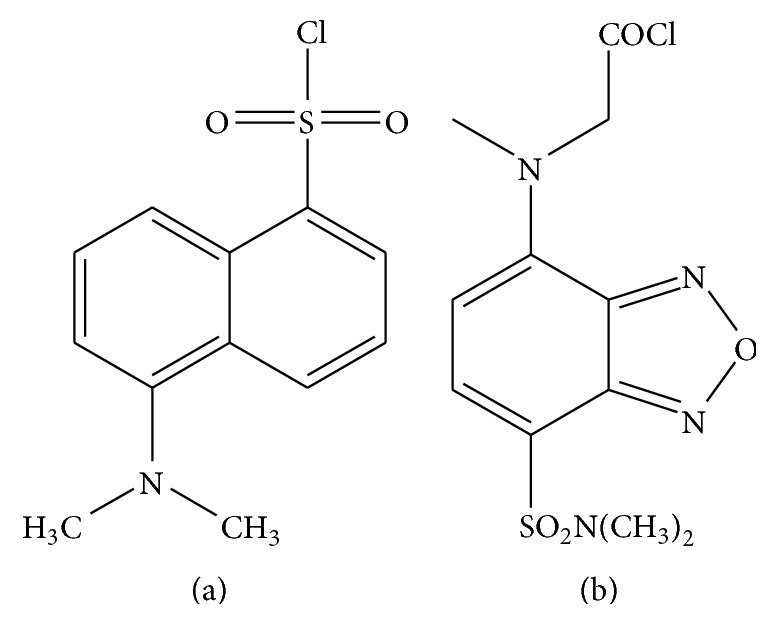
Structures of common derivatizing agents used in HPLC with UV or fluorescence detectors. (a) Dansyl chloride. (B) 4-(*N*-Chloroformylmethyl-*N*-methyl) amino-7-*N*,*N*-dimethylaminosulphonyl-2,1,3-benzoxadiazole (DBD-COCl).

There are several methods describing chromatographic separation of endocannabinoid metabolites by HPLC coupled with UV or fluorescence detectors. Due to the chemical structures of these lipid molecules which lack of chromophores or fluorophores, chemical derivatization is required prior to the analysis. Yagen and Burstein described a method for detection of AEA as its dansyl derivative. Dansyl esters of AEA can be generated by heating AEA with excess dansyl chloride (Figure 5) and dimethylaminopyridine in acetone at 55°C for 1 hr. The product can be separated by silica gel TLC plate using chloroform : hexane : methanol (90 : 100 : 3 v/v) and visualized by UV light at 365 nm [51]. Alternatively, Wang et al. used 4-(*N*-chloroformylmethyl-*N*-methyl) amino-7-*N*,*N*-dimethylaminosulphonyl-2,1,3-benzoxadiazole (DBD-COCl; Figure 5) to derivatize AEA and 2-AG. The DBD-CO derivatives of AEA and 2-AG were subsequently subjected to separation by HPLC and identified with a fluorescence detector set to 450 nm excitation and 560 nm emission wavelengths [86].

#### 3.2.2. LC-MS and LC-MS/MS

In LC-based MS methods, endocannabinoid metabolites can be analyzed directly by soft ionization techniques including electrospray ionization (ESI) [6, 48] and atmospheric pressure chemical ionization (APCI) [68, 87]. LC-MS approaches usually utilize SIM mode to select the molecular ions ([M + H]^+^), sodium adducts ([M + Na]^+^), or potassium adducts ([M + K]^+^) in positive ion mode [52, 53]. Some groups also have used negative ion mode for detection of endocannabinoid metabolites [61, 88]. In addition to combinations of mobile phase solvents mentioned above for separation, ammonium acetate is frequently added to the mobile phase which will generate ammonium adducts ([M + NH_4_]^+^) by ESI-MS in positive mode [54, 57]. Although LC-MS approaches using SIM mode have been efficiently used to quantify endocannabinoid levels, the popularity of tandem mass spectrometry (MS/MS) has increased over the years due to the higher selectivity and additional structural information. Selected reaction monitoring (SRM) mode after ESI typically is achieved by a triple-quadrupole MS to select ions of interest and identify the fragmentation products. This process can enhance sensitivity, reduce background noise, and increase signal to noise ratio. The same SRM methodology may be also applied to GC-MS-based analysis.

*(1) AEA*. In positive-mode ESI-MS, AEA is found as a protonated molecule [M + H]^+^ [57] at* m/z* 348, as an adduct with sodium [M + Na]^+^ at* m/z* 370, and/or as an adduct with potassium [M + K]^+^ at* m/z* 386. When [M + H]^+^ is subjected to CID, the mass transition of* m/z* 348 → 62 is formed due to loss of ethanolamine moiety [57]. Formic acid can be added as part of mobile phase to increase the yield of [M + H]^+^. The concentration of formic acid is usually in the range of 0.05–0.25% v/v [57, 62, 89]. Although ESI-MS in positive mode is more popular that ESI-MS in negative mode, some groups performed the analysis with the negative mode where [M − H]^−^ ions at* m/z* 346 are observed [61].

Several groups have added silver acetate (approximately 70 *μ*M) to the mobile phase to increase the sensitivity of detection for AEA in LC/MS/MS analysis. Adducts with silver ions are observed as two [M + Ag]^+^ adducts due to the isotope distribution of silver which is ^107^Ag (52%) and ^109^Ag (48%). [M + ^107^Ag]^+^ and [M + ^109^Ag]^+^ correspond to* m/z* 454 and 546, respectively. When [M + ^107^Ag]^+^ is subjected to CID, the mass transition* m/z* 454 → 436 is formed due to loss of water [53, 90].

*(2) 2-AG*. When analyzed in positive-mode ESI-MS, 2-AG typically yields the protonated molecule [M + H]^+^ at* m/z* 379 which is usually analyzed by LC/MS analysis. 2-AG also forms adducts with sodium [M + Na]^+^ and potassium [M + K]^+^ which yield ions at* m/z* at 401 and 417, respectively. If ammonium ions are present in the mobile phase, the ammonium adducts [H + NH_4_]^+^ can be observed at* m/z* 396 [57]. Under CID, the fragment ion of* m/z* 361 can also be seen as a loss of water [M + H − H_2_O]^+^ [48]. A loss of glycerol [M − C_3_H_8_O_3_]^+^ corresponds to fragment ion at* m/z* 287 [91]. 2-AG also may form adducts with silver ions in the presence of silver acetate in mobile phase. The molecular ion generated from positive mode ESI is [M + ^107^Ag]^+^ observed at* m/z* 485 for ^107^Ag isotope. CID of* m/z* 485 yields mass transition of* m/z* 485 → 411 due to loss of glycerol [90].

**Figure 6:**
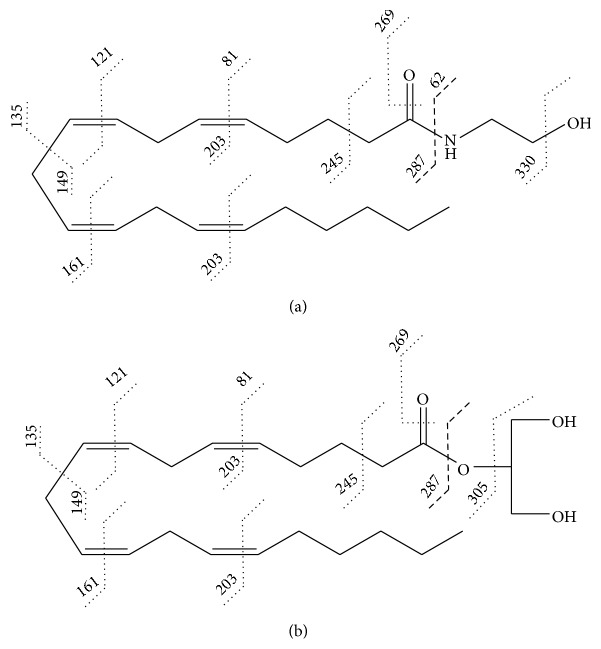
Fragmentations from LC/MS/MS in positive ESI mode of (a) [AEA + H]^+^ at* m/z* 348.4 and (b) [2-AG + H]^+^ at* m/z* 379.2 after CID [19].

Fragmentation schemes for [AEA + H]^+^ at* m/z* 348.4 and [2-AG + H]^+^ at* m/z* 379.2 after collision induced dissociation (CID) are illustrated in Figure 6 [52].

*(3) Isomerization of 2-AG to 1-AG*. Because 2-AG spontaneously undergoes isomerization to biologically inactive 1-AG through the migration of acyl group from the* sn*-2 to* sn*-1/3 position as observed in several studies [47, 72, 92], 2-AG should not be analyzed without taking into consideration of 1/3-AG. Zoerner et al. tested the effect of several solvents on isomerization of 2-AG to 1/3-AG and showed that no isomerization was observed when toluene was used as a solvent [88]. Further, the ratio of 2-AG/1-AG was 12.3 : 1, 10.8 : 1, 9.8 : 1, 9.5 : 1, 8.1 : 1, 7.5 : 1, 6.9 : 1, 4.4 : 1, 4.1 : 1, 2.6 : 1, and 0.07 : 1 for chloroform, isohexane, ethyl acetate, ethyl acetate-heptane, water, ethyl acetate:isohexane, acetonitrile, chloroform:methanol, acetone, methanol, and ethanol, respectively. Thus, solutions of 2-AG in methanol or ethanol should be avoided during sample preparation to prevent the acyl migration.

## 4. Example of Routine NAEs Analysis by GC-MS

**Figure 7:**
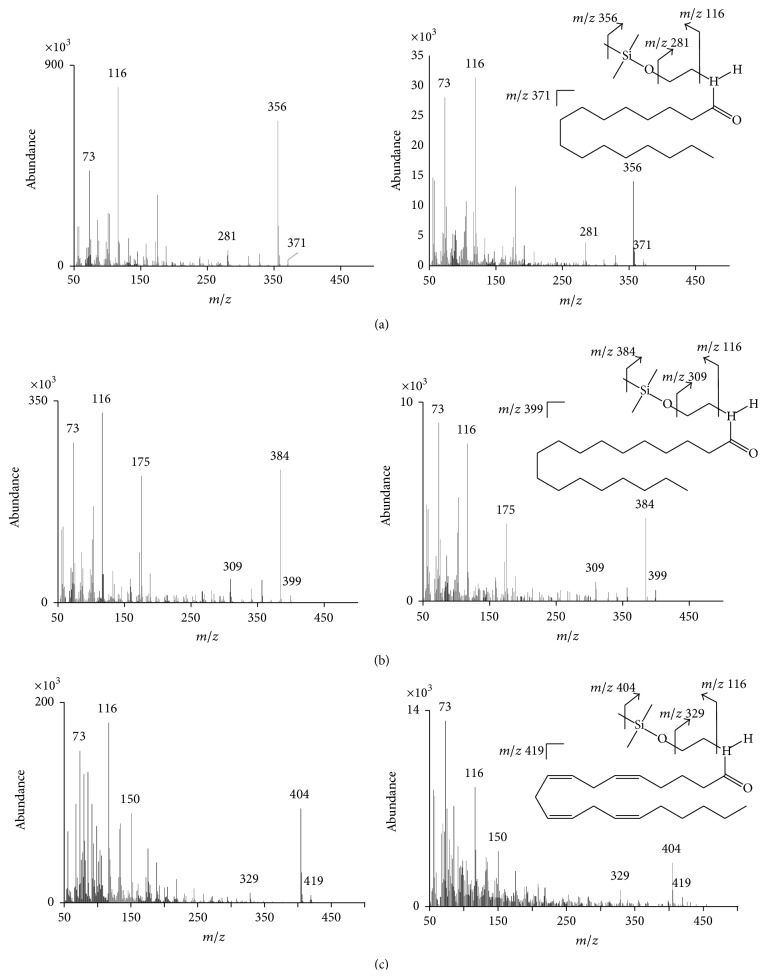
Mass spectra of NAEs generated by GC-MS with positive EI in scan mode. (a)* N*-Palmitoylethanolamine (PEA). (b)* N*-Stearoylethanolamine (SEA). (c)* N*-Arachidonoylethanolamide (AEA). (Left column represents authentic, purified compounds; right column represents the same compounds identified in retinal tissue extracts.)

By way of example, the following procedure is suitable for the analysis of endocannabinoid-type metabolites from a variety of biological tissues. Extreme care should be taken to minimize postmortem accumulation of endocannabinoid metabolites. Frozen or fresh tissues (50–100 mg per sample) are homogenized in a bead beater apparatus in hot 2-propanol (2.5 mm dia glass beads; Bio Spec Products Inc.). The extract is combined with deuterated NAE standards (d4-NAE 16 : 0, d4 NAE 18 : 2, and d4 NAE 20 : 4) (Cayman Chemical Co.; 100 ng each) and total lipids are extracted into chloroform. The organic phase is collected for further purification by solid phase extraction (SPE). Silica SPE cartridges (100 mg, 1.5 mL; Grace Davison Discovery Sciences) are conditioned with 2 mL methanol followed by 4 mL chloroform. Samples are applied to the SPE column and washed with 2 mL chloroform, and NAEs are eluted with 2 mL of 1 : 1 (v/v) ethyl acetate:acetone. The eluate is collected, evaporated under nitrogen, and derivatized with 50 *μ*L BSTFA (Fisher Scientific, Houston, TX, USA) for 30 min at 55°C. After derivatization, the samples are again evaporated under nitrogen and reconstituted in 50 *μ*L hexane. NAEs are identified via selective ion monitoring and quantified against the internal deuterated standards (d4- NAE 16 : 0) as TMS-ether derivatives by GC-MS (Agilent model 6890 GC coupled with a 5973 mass selective detector) in positive EI. NAE concentration is then calculated based on fresh weight, dry weight, or lipid weight. Concentrations of several NAEs in serum, heart, brain, and retina of 6-week-old male DBA/2 mice before and after depot injection of NAE 16 : 0 were determined by this method and reported in Jian et al. [85]. The NAEs species detected were NAE 16 : 0, NAE 18 : 0, NAE 18 : 1, NAE 18 : 2, and NAE 20 : 4 (AEA). Representative mass spectra of selected NAEs analyzed by GC-MS with EI-MS in positive scan mode are shown in Figure 7.

## 5. Construction of Standard Curves

Standard curves (calibration curves) for both GC-based and LC-based analyses are typically constructed by combining a constant amount of internal standard(s) (IS) with increasing amounts of the corresponding native molecules (analytes). Concentrations of analytes in biological samples can be determined by calculating of their responses against internal standard responses as described in [54]. One important precaution to consider is that deuterated standards may contain some parental, unlabeled compound and consequently should be run through analytical procedures in the absence of tissue to account for this potential contribution especially where analytes are in very low abundance:

(1) Relative  response=peak  area  (or  ion  abundance)  of  analytes(IS  area/IS  concentration).

## 6. Summary and Discussion

Advances in the development of analytical methodologies for endocannabinoid and endocannabinoid-like compounds in biological tissues and fluids have helped to support further understanding of how these lipid mediators function in a variety of organisms and biological processes [10, 22]. The functions of these metabolites often rely on rapid changes in their endogenous concentrations and so it is imperative that accurate, sensitive procedures be employed to assess their identities and quantitative abundances [45, 52]. Various mass spectrometric approaches fit these requirements well, and while standard GC-MS procedures have been in place the longest [47, 63], LC-MS and tandem MS provide for separation, sensitive detection, and structural identification of samples and are especially applicable to underivatized metabolites [19, 46, 52, 61]. In both MS approaches, the most accurate quantification is based on robust isotope dilution procedures and referenced to standard curves for respective analytes.

The analysis of endocannabinoid and endocannabinoid-like compounds can be a challenging goal, especially given their low concentrations in biological organisms [45]. As pointed out elsewhere [19, 45, 47, 52], great care should be taken in the handling of tissues and extracts so that identification and quantification of endogenous metabolites are as accurate as possible. Sample preparation is one of the most important steps to consider due to the low abundance and potential for degradation or postmortem production during tissue processing [63]. One additional complication for 2-AG analysis is the spontaneous isomerization of 2-AG to 1/3-AG. The chromatographic separation between these two isomers is necessary due to the similarity of how they behave during MS or MS/MS analysis [10]. Additional precautions should also be taken into consideration throughout tissue handing, homogenization, and lipid extraction. Tissues should be stored at −80°C and not allowed to thaw until they are extracted. The exposure of lipid samples to plastic should be minimized to avoid losses during processing or extraction of contaminants from surfaces that may interfere with analyses. Of course minimal exposure to light and oxygen is necessary to maintain integrity of metabolites as well. Most important is the use of internal standards that help to account for losses during the analysis and facilitate identification and accurate quantification.

While the targeted MS-based lipidomics approaches described herein are valuable for quantification of endocannabinoids, they are also suitable for untargeted applications and the discovery of novel lipid metabolites with potential functional significance. These analytical technologies support an exciting time in biomedical research where the list of functional lipid mediators is growing at an amazing pace. In the case of endocannabinoids and endocannabinoid-like molecules, their analyses most certainly will continue to provide clues for lead therapeutic agents or for biomarkers of various pathological conditions.
